# Validation of near infrared fluorescence (NIRF) probes *in vivo* with dual laser NIRF endoscope

**DOI:** 10.1371/journal.pone.0206568

**Published:** 2018-11-02

**Authors:** Manisha Shrivastav, Elias Gounaris, Mohammad W. Khan, Jeffrey Ko, Stacy H. Ryu, Matthew Bogyo, Andrew Larson, Terrence A. Barret, David J. Bentrem

**Affiliations:** 1 Department of Surgery, Feinberg School of Medicine, Northwestern University, Chicago, Illinois, United States of America; 2 R.H. Lurie, Comprehensive Cancer Center, Northwestern University, Chicago, Illinois, United States of America; 3 Gastroenterology Division, Feinberg School of Medicine, Northwestern University, Chicago, Illinois, United States of America; 4 Department of Pathology, Stanford University, School of Medicine, Stanford, California, United States of America; 5 Department of Radiology, Feinberg School of Medicine, Northwestern University, Chicago, Illinois, United States of America; 6 Department of Internal Medicine, Medical School, Kentucky University, Lexington, Kentucky, United States of America; Massachusetts General Hospital, UNITED STATES

## Abstract

**Purpose:**

The development of NIRF cathepsin activity probes offered the ability to visualize tumor associated tumor reaction and act as a surrogate marker to delineate the dysplastic lesions. One major type is a NIRF substrate of cathepsins (SBP), which is involved in catalytic way to produce high levels of fluorescence emission. The other major type (ABP) reacts with active cathepsins in stoichiometric manner since they bind covalently with their active center. Little is known about the sensitivity and the specificity of the NIRF probes to detect autochthonous developed dysplastic lesions. Dual laser NIRF endoscope provides a good tool to determine the efficiency of various NIRF probes *in vivo* in the same lesions.

**Experimental design:**

In the current study, we validated both types of NIRF probes by using the dual laser NIRF endoscope to detect lesions colon cancer mouse model (TS4Cre/cAPC ^+/lox^).

**Results:**

The dual laser NIRF endoscope is emitting equal power with both lasers. It can detect with the same efficiency in 680 mode, as well as, 750 mode when NIFR probes of the same scaffold *in vivo*. When SBP and ABP were used, our results showed both probes are efficient enough to detect large polyps but small dysplastic lesions could not efficiently imaged with the ABP.

**Conclusions:**

The dual laser NIRF endoscope is a powerful tool to validate probes. The probes that react catalytically with the active center of cathepsins are more efficient than the ones that react stoichiometrically in detecting small lesions.

## Introduction

Early detection of dysplastic lesions is important for the effective colon cancer diagnosis and treatment [1,2]. Over the past few decades, improvements in endoscopic technology have enabled earlier therapeutic interventions [3] thus achieving a marked reduction in the overall incidence of colon cancer [4,5]. However, colon cancer remains the most commonly diagnosed cancer in the western world.

Recent efforts have sought to develop more sensitive and specific detection approaches wherein lesions are detected after the introduction of fluorescent probes that exhibit either a specific affinity to colon cancer cells (octa-peptides tagged with fluorescein in human studies or Cy5.5 for studies in mice [6] [7]) or selective activation based upon biological activities specifically elevated within dysplastic and/or neoplastic tissues. Probes that detect biological activity associated with neoplastic, pre-neoplastic and acute inflammatory lesions were designed to have the following properties: A) to be able to specifically register cancer associated biological activity, B) to produce a high signal to noise ratio, C) to be visible even when the lesions are submerged deep in the tissue (intraepithelial neoplastic lesions). Almost all probes developed are emitting in the near-infrared fluorescence (NIRF) spectrum. In this part of the spectrum, the auto-fluorescence of the tissue is minimal, and the long wavelength of the fluorochromes is supporting the deeper penetration of the fluorescent light through the tissue.

Cysteine cathepsin activity probes are the most common agents used [8]. Cysteine cathepsins are lysosomal proteolytic enzymes. They are distributed in infiltrating innate immune cells[9]. Tumor-associated inflammation, sustained by innate immune cells, is required to support the early cancerous lesions by providing the required growth factors and contributing to the neo-vascular development [10]. The detection of dysplastic lesions with the use of NIRF probes is proven to be sensitive and specific both *in vivo* when administered in murine polyposis models [11] and *ex vivo* [10].

There are two main types of NIRF probes suitable for detection of cathepsin activity: A) the substrate-based probes (SBP) and B) activity-based probes (ABP). SBPs mimic substrates of cathepsin proteolytic activity, having a backbone of poly-L-Lysine [9]. Connected to the ε-amino group of the L-lysine moiety are fluorochromes at a concentration high enough to quench each other’s fluorescence emissions. The liberated proteolytic products are optically unquenched [12] and emit in the near infrared spectrum (NIRF). Activation of cysteine cathepsins activity probes in the tissue would mean more fluorescent product formation that emits more fluorescence from the tissue. ProSense 680 and 750 are commercially available non-specific substrates of a broad range of different cathepsins.

Cathepsin-specific NIRF SBPs share the same poly-L-lysine backbone but the fluorophores are linked to a peptide spacer that contains the amino acid sequence that is recognized and hydrolyzed by specific cathepsins. There are SBPs of this form for cathepsin B and cathepsin K [13,14].

The mechanism of action of ABPs resemble to suicide inhibitor. When ABPs bind to the active center of an active cathepsin, a stable bond is formed with the enzyme (function is stoichiometric with one probe binding to one active enzyme). Most of these probes are un-quenched, although there are some quenched probes developed recently [15]. Some cathepsin-specific ABPs for specific cathepsins are available (mostly cathepsin S and B) [15].

Very little has been reported on the sensitivity and specificity of the NIRF cathepsin activity probes. The probes were compared using mice with implanted tumors of uniform size [16]. This technology can become translational only if, the effectiveness of the probes to detect lesions of various size throughout the tissue can be demonstrated.

We demonstrated that NIRF SBPs can detect *in vivo* polyps with high sensitivity and specificity. An invasive intravital microscopy method was employed, using transgenic polyposis mouse models APC^Δ468^. [10]. Recently we reported the development of a single-channel NIRF endoscopic system, able to discriminate IL-10 colitis-induced dysplasia from colitis in murine colitis models [11]. For the latter studies, we used a NIRF endoscope with excitation light produced from a Xenon light source (excitation at 680nm after spectral separation) and ProSense 680 cathepsin probes[17]. This endoscopic system was able to detect polypoid, intraepithelial flat lesions, colitis, and lymphoid aggregations *in vivo*, in a colitis mouse model (IL10^-/-^ Piroxicam) [18]. Only one report has been published comparing SBP with ABP in animals with injected tumor cells and *in vitro* detection [19].

In an effort to rigorously compare co-registered *in vivo* studies and evaluate for salient combinations of different NIRF probes during a single examination, we recently developed a new dual laser multi-channel NIRF endoscope. Herein we report the development and systemic evaluation of this new NIRF endoscope along with initial studies using this dual laser endoscope for comparisons of probes in the same lesions in a transgenic mouse model of colon cancer *in vivo*.

## Materials and methods

### Ethics statement

This study was carried out in strict accordance with the recommendations in the Guide for the Care and Use of Laboratory Animals of the National Institutes of Health. The protocol was approved by the Institutional Animal Care and Use Committee (**IACUC**) of Northwestern university (Protocol Number: IS00003036). Throughout the in vivo procedures, mice were anaesthetized with inhalation of 1.5–2% mixture of isoflurane in oxygen (1L/hour) according to the approved CCM protocol. After the endoscopy, mice were euthanized while in deep anesthesia level with cervical dislocation, and the colon was examined with reflectance fluorescence and histology. The euthanasia procedure was approved by the Center of Comparative Medicine of the Northwestern University.

### Experimental animals

All animals used for these studies were housed in the barrier facility of the Center for Comparative Medicine (CCM) at Northwestern University, Chicago Illinois, and the procedures described were performed with approval of CCM and IACUC committees Northwestern University. All animal work was approved and conducted under the guidelines of Northwestern University’s Animal Care and Use Committee. The barrier facility of Northwestern University at is providing clean environment with normal cycles of light and darkness, normal chaw and water as well environmental enhancement. Mice that develop colon polyps are inspected every second day. Mice with signs of distress and prolapse were euthanized immediately. Nevertheless, mice younger than 5 months were used as experimental group.

### Dual laser NIRF endoscope

A dual laser near infrared fluorescence endoscope was developed in collaboration with Olympus (Tokyo, Japan). The intended purpose of this system was to permit *in vivo*, comparison studies using multiple NIRF cathepsin probes or simultaneous *in vivo* interrogation of multiple biological processes. This system was intended to: A) permit two independent examination modes (namely at 680 nm and 750 nm wavelengths), B) have minimal cross talk between the two examination modes, C) produce well-adjusted excitation light energy such that the two examination modes would be evenly weighted, D) operate with a number of fiberscopes with diverse specifications, and finally E) permit image recording at video frequency (10 frames/second).

### Laser excitation light source

**Fig 1:**
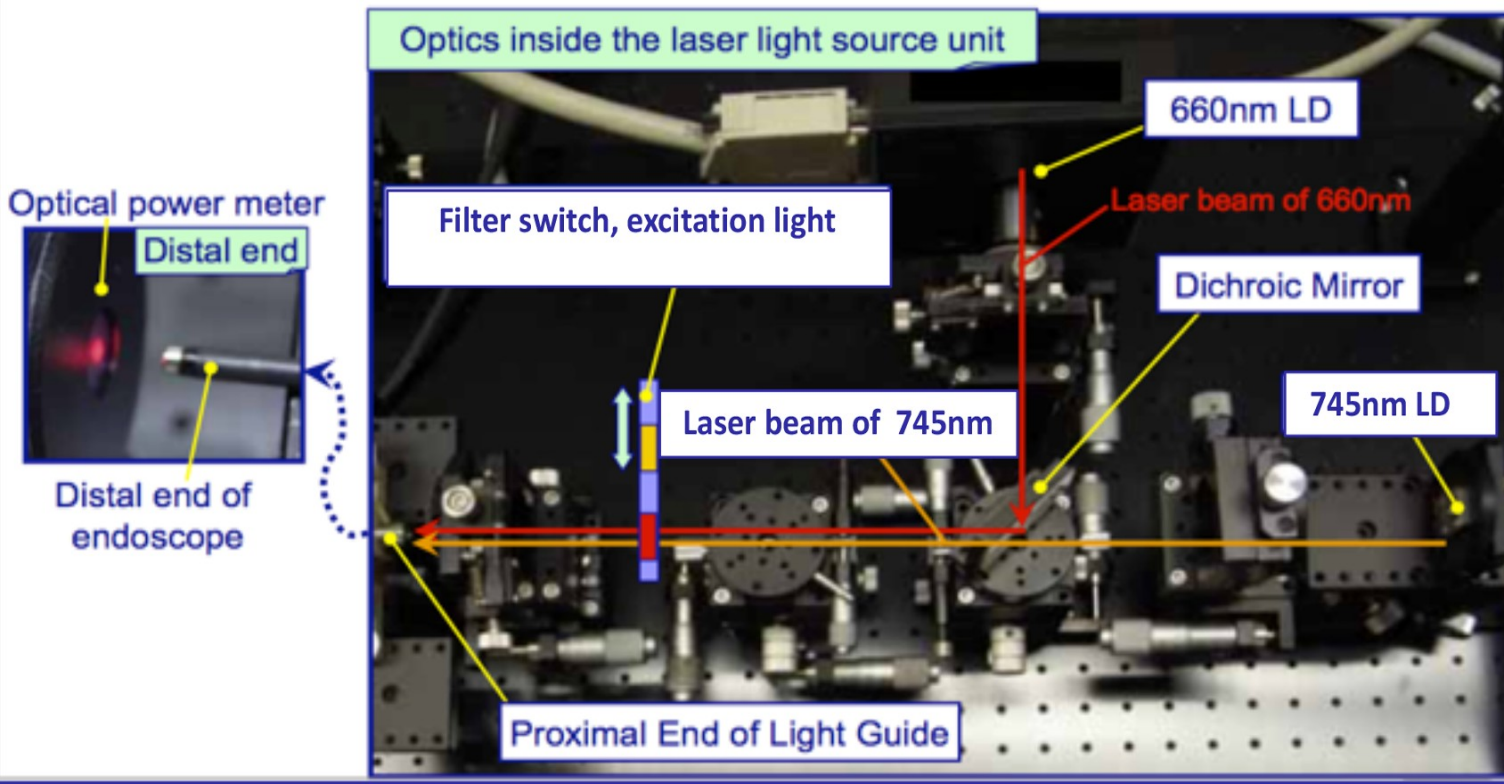
Optics inside the laser excitation light source unit. Yellow pointers are indicating the two lasers, the dichroic filter and the filter switch. The light path of 660nm is illustrated with red arrows and the 745nm path is illustrated with brown arrows.

The excitation system consists of two lasers (660 nm and 747 nm). The power output of each laser is controlled externally. Two external thermal regulators used to regulate the temperature of the lasers. The excitation beams are focused upon a dichroic mirror forming a 90^o^ angle such that, the parallel beams can be filtered. The excitation light filtering system consists of two band-pass filters, 400-680nm and 720-750nm. The bandwidth of these filters was selected to limit spectral overlap. These two filters are on a carousel that permits the user to interchange filters to alter the laser’s output (Fig 1). The filtered light is passing into the fiberscope through an Olympus universal connector allowing users to connect a broad array of Olympus fiberscopes with various different specifications.

### Fiberscopes

Current configuration permits connection of A) pancreatic duct fiberscope (PF-8P) which is flexible but cannot bend actively and has a very narrow insertion tube diameter (0.8 mm), B) neonatal bronchoscope (BF-N20) with active bending section, and a relatively small (2.2mm) insert tube diameter, and C) neonatal bronchoscope (BF-XP60) with active bending section and an instrument channel (used during our mouse studies to insufflate colon, wash, collect biopsies and perform injections). All fiberscopes were equipped with a wide-angle lens on the tip (75^o^ for PF-8P & BF-N20) and extending to 90^o^ for BF-XP60 (S1 Fig). Wide-angle lenses decrease the spatial resolution of the optical system, but this is a necessary trade-off for NIRF endoscopy because it is important in order to detect focal fluorescence emission within deep focal planes. We used the BF-XP60 for our experiments.

### Image detection and recording

The image detection of fluorescence emissions is similar to the system already published [11]. In short, the fluorescence emission light coming from the fiberscope is split with a dichroic mirror. The fluorescence reflectance is detected with a CCD camera and integrated in an image integrator with the fluorescent light image that is detect by a high sensitivity EM-CCD camera.

Software is used to synchronize the two images and display each in real time on video monitor (these images are also stored at a rate of 10 frames per second). The fluorescent light images are filtered through a set of band-pass emission filters (680–720 nm and 720-750nm) to avoid cross talk between channels. These emission filters are located on a carousel and can be interchanged manually. This dual laser NIRF endoscopy system permits two modes of examination: the **680 mode** (660 nm laser ON, 400–680 nm excitation filter and 680–720 nm emission filter) and the **750 mode** (730 nm laser ON, 720–750 nm excitation filter and 765–850 nm emission filter). Each frame image is saved as Olympus tiff 16bit files. These are stored in a folder named according to the date and the time of the experiment. The collected images can be analyzed with either the endoscope software or with Image J using the LOCI plugin for import. All tiff files can be converted into video files by using Image J.

### Assessment of laser power

A portable light power meter was used to calculate the power of the lasers at the tip of the fiberscope. The power meter was equipped with a sensor. The tip of the fiberscope, BF-XP60, was 0.5 cm from the sensor. The power was measured for both 680 and 750 modes.

### *In vitro* Cross talk analysis

To access the possible cross talk between the channels, serial dilutions of the fluorochromes Cy5.5 and Cy7.0 were set in 100 μl capillary tubes closed by heating at both ends. The capillary tubes with various fluorophore concentrations were placed upon a black background to avoid any reflection of the laser light. Capillaries were imaged with both 680 and 750 modes setting the distance of the tip at 3, 11, and 50 mm. The mean fluorescence intensity (MFI) of the recorded images was calculated with the use of Image J software and plotted with the GraphPad Prism software.

### *In vivo* cross talk analysis

Mice were injected retro-orbitally with 100 μl of either AngioSense 680 or AngioSense 750 (purchased from VisEn Medical). AngioSense is a relatively large homo-polymer that is linked with either Cy5.5 or Cy7.0. AngioSense remains in the vessels, and because it is large it diffuses slowly from the vessels into the tissues. Ten minutes after the injection, the mice were anesthetized with isoflurane and the subcutaneous vessels close to inguinal lymph nodes were exposed. These vessels were imaged in both 680 and 750 modes regardless of the type of AngioSense injected thus, allowing cross talk comparisons.

### NIRF probes administration

ProSense 680 and 750 (both substrates based cathepsin activity probes) were purchased from VisEn Medical. GB138 (an activity based cathepsin probe) was provided by generous gift of Dr. Matthew Bogyo, Stanford University. ProSense probes are peptides readily soluble in aqueous solutions. Twenty-four hours before the imaging session, 2 nmoles of these ProSense probes were injected retro-orbitally. GB138 requires 20–40% of organic solvents (DMSO) to remain in solution. Thus, GB138 requires slow infusion rates in the tail vein. In our experiments we used 25 nmoles of GB138 per mouse as recommended[15,20].

### Endoscopic examination

Mice were injected with 2 nmoles of SBP[17], and/or 25 nmoles of ABPs 24 [21] hours before the endoscopy session. The colon of the anesthetized mice was washed with 5 ml phosphate-buffered saline (PBS) delivered with either a non-traumatic needle with blunt end or by using the BF-X60 instrument channel. PBS acts as a lubricant to facilitate the fiberscope insertion. The insertion of the two fiberscopes, BF-XP60 and BF-N20, was aided by the use of their active bending tips. With the BF-XP60 we can carefully insufflate the colon for convenient observation. During these *in vivo* imaging procedures, the insertion distance of the fiberscope was noted whenever, a lesion was observed; these measurements allowed us to co-localize these lesions at colon necropsy. [17]

### Histopathological studies

The mouse colons after endoscopy were excised and flayed open. After reflectance fluorescence of the whole mount the colons were Swiss rolled and frozen in OCT. The blocks were sectioned until completion in 5μm section. Every tenth section were stained with a modification of H&E[22]. In short sections were washed with PBS, and stained with Hematoxylin solution (Mayer’s) for 3 min. The sections were placed under running tab water for at least 5 min. After this wash samples were stained in eosin Y solution for 2 min. After dehydration (95% ethanol, 95% ethanol, 100% ethanol 2min, 3 changes in xylene for 2 min per change). The sections were mount on a drop of Permount over the tissue and coverslip. Slides were viewed under light microscope.

### Signal to noise ratio (SNR)

We calculated the mean fluorescent intensity (MFI) of three regions of interest (ROI) per frame. ROIs were defined with the use 30^2^ pixels square and the MFI was calculated with Image J software. One ROI (ROI 1, lesion) was located on the brightest point of the frame, the second (ROI 2, tissue noise) on the darkest point inside the tissue and the third (ROI 3, camera noise) on the frame out that surrounds the image.

The MFI of each ROI was calculated with the Image J software. The SNR is calculated according to the formula {ROI1 –ROI3}/ {ROI2 –ROI3}. The results are plotted and analyzed statistically with the GraphPad Prism 5 software.

### Statistical analysis

Statistical analysis of the experimental values, expressed as [Mean value ± SEM], is performed with t test.

## Results

### The power output of the laser

When the power of laser is in the highest settings, we can measure 30–35 mW from 660 nm and 45 mw from 745 lasers at the tip of the BF-XP60 fiberscope. We calculated the light energy released from the tip of the fiberscope. The lasers were on their highest settings. The sensor of a power meter was set 0.5 cm from the tip of the BF-XP60 fiberscope. We calculated 35 mW using the 680 mode and 45 mW using the 750 mode.

### The cross talk between the channels

The fluorescence endoscopy detection is determined by the following parameters: A) the concentration of the fluorochromes, B) the distance of the object from the tip of the fiberscope, and C) the use of specific filters.

To determine the minimal concentration needed to visualize the fluorophores, the maximal distance from the tip of the fiberscope phantoms either in the optimal mode (680 nm laser for Cy5.5 and 750 nm for Cy7.0) at various concentrations (10^1^, 5x10^1^, 10^2^, 5x10^2^, 10^3^ and10^4^ nM) at 3 mm, 11 mm and 50 mm distance.

**Fig 2:**
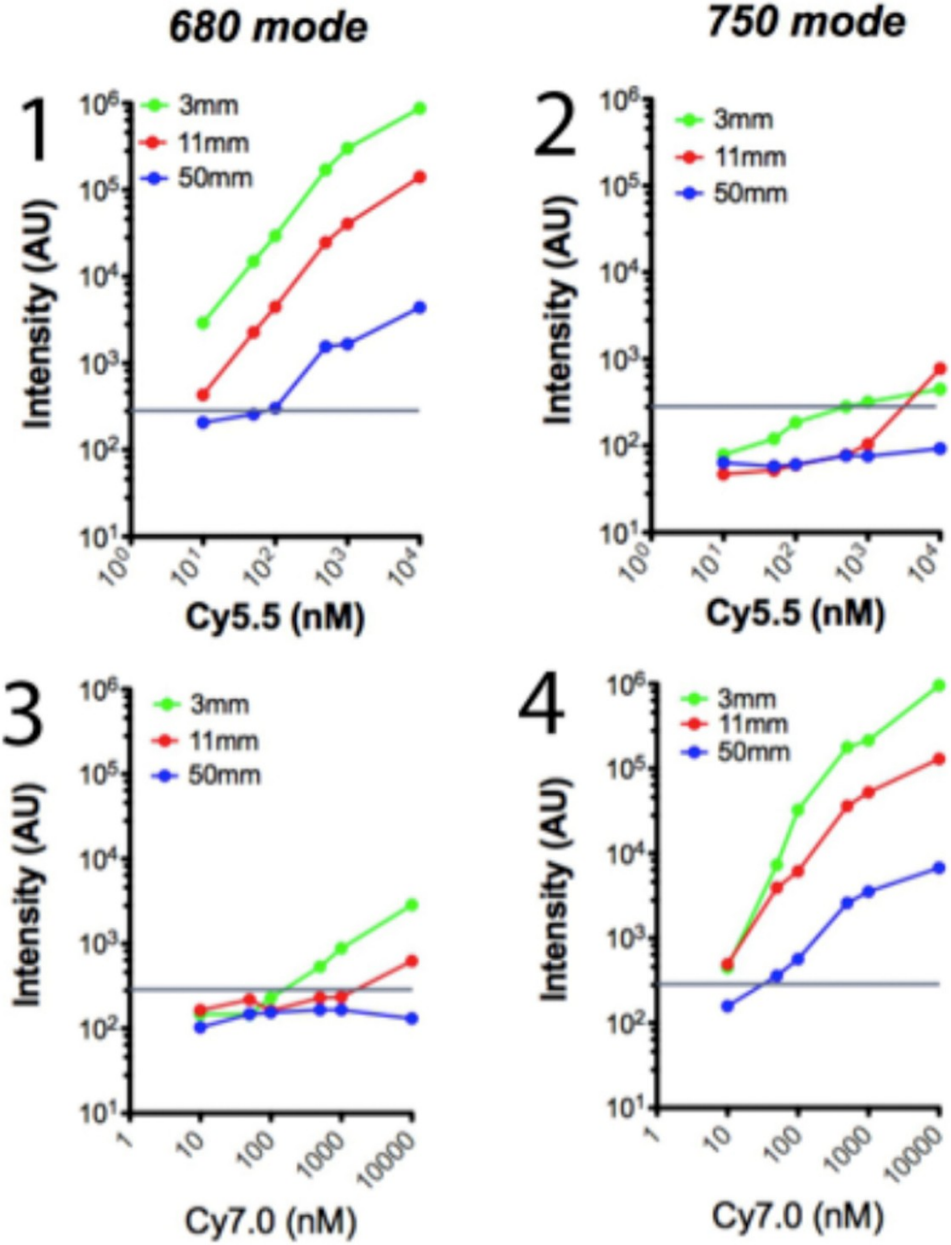
Analysis of cross talk of the two lasers *In vitro*. Phantoms have been created containing 10^1^, 5x10^1^, 10^2^, 5x10^2^, 10^3^ and10^4^ nM of fluorophore Cy 5.5 (n = 6) and Cy 7.0 (n = 6). All phantoms were examined with the fiberscope tip being at 3mm (green dots and lines), 11mm (red dots and lines) and 50mm (blue dots and lines) The Cy 5.5 phantoms were examined with the 680 mode (Fig 2, 1) and 750 mode (Fig 2, 2), and the Cy 7.0 were examined with 680 mode (Fig 2, 3) and 750 mode (Fig 2, 4). The images of the phantoms were recorded and their image intensity were calculated with image J and recorded as intensity arbitrary units (AU). The horizontal line represents the electronic noise of the endoscopy system.

To determine the fluorochrome concentrations above which it can be visible utilizing the optimal mode, we prepared phantoms that contained increased concentrations of the fluorochromes Cy5.5 (Ex/Em 675/695) and Cy7 (Ex/Em 743/767), and we observed them with both 680 and 750 mode placing the tip of the endoscope 3 mm 11 mm and 50 mm from the phantoms; we calculated the intensity of the phantom’s images in arbitrary units as analyzed with Image J **(**Fig 2).

The Cy5.5 and Cy7.0 phantom imaging revealed that fluorochromes can be detected when excited with the corresponding examination mode even in low concentrations with the tip of the fiberscope as far as 50 mm from the phantoms. When the phantoms were imaged in the opposite mode, namely 750 mode for Cy5.5 and 680 mode for Cy7.0, the signal observed was minimal and only in close observation (3 mm), and in high fluorochrome concentration (10^3^ to 10^4^ nM). It should be noted that the plot axes in Fig 2 are logarithmic.

**Fig 3:**
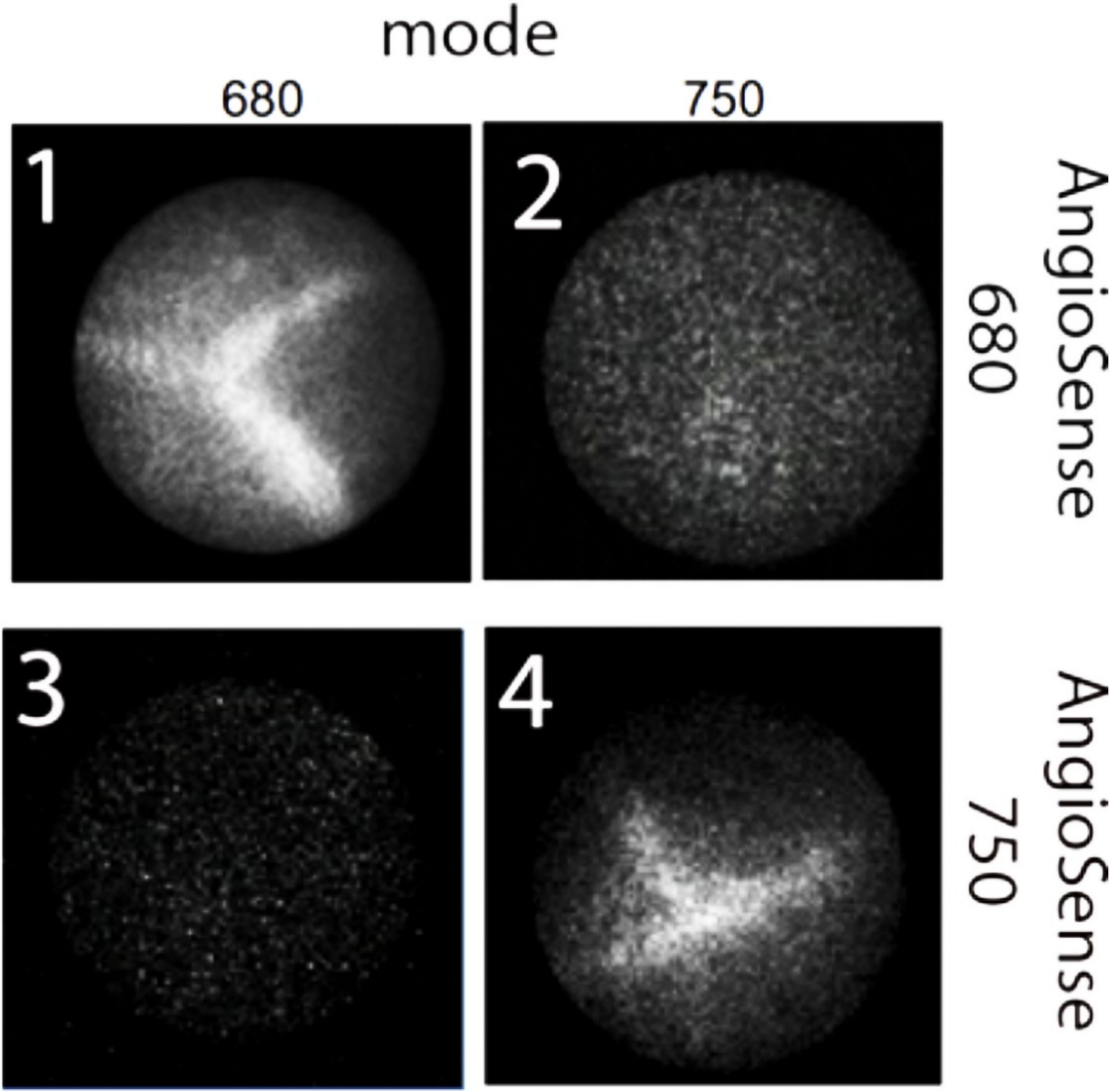
Representative images of vessels injected with AngioSense 680 (1&2) or AngioSense 750 (3&4) All mice were either imaged with 680 mode (1&3) and 750 mode (2&4).

To verify the *in vitro* results, we imaged vessels *in vivo* using mice injected with vessel contrasting agents AngioSense 680 and AngioSense 750 in both 680 and 750 modes (Fig 3). In the *in vivo* experiments, mice injected with AngioSense 680 showed major vessels using 680 mode while there was no signal when imaged in 750 mode (Fig 3, panels 1&2). Mice injected with AngioSense 750 show signal when imaged in 750 mode (Fig 3, panel 4), and no signal at all in 680 mode imaging (Fig 3, panel 3). Observation was done 10 mm from the tissue. It is clear that at concentrations of probes used in *vivo* the cross talk between the two modes of imaging is negligible.

### NIRF endoscopy can detect polyps with equal efficiency using 680 and 750 mode

**Fig 4:**
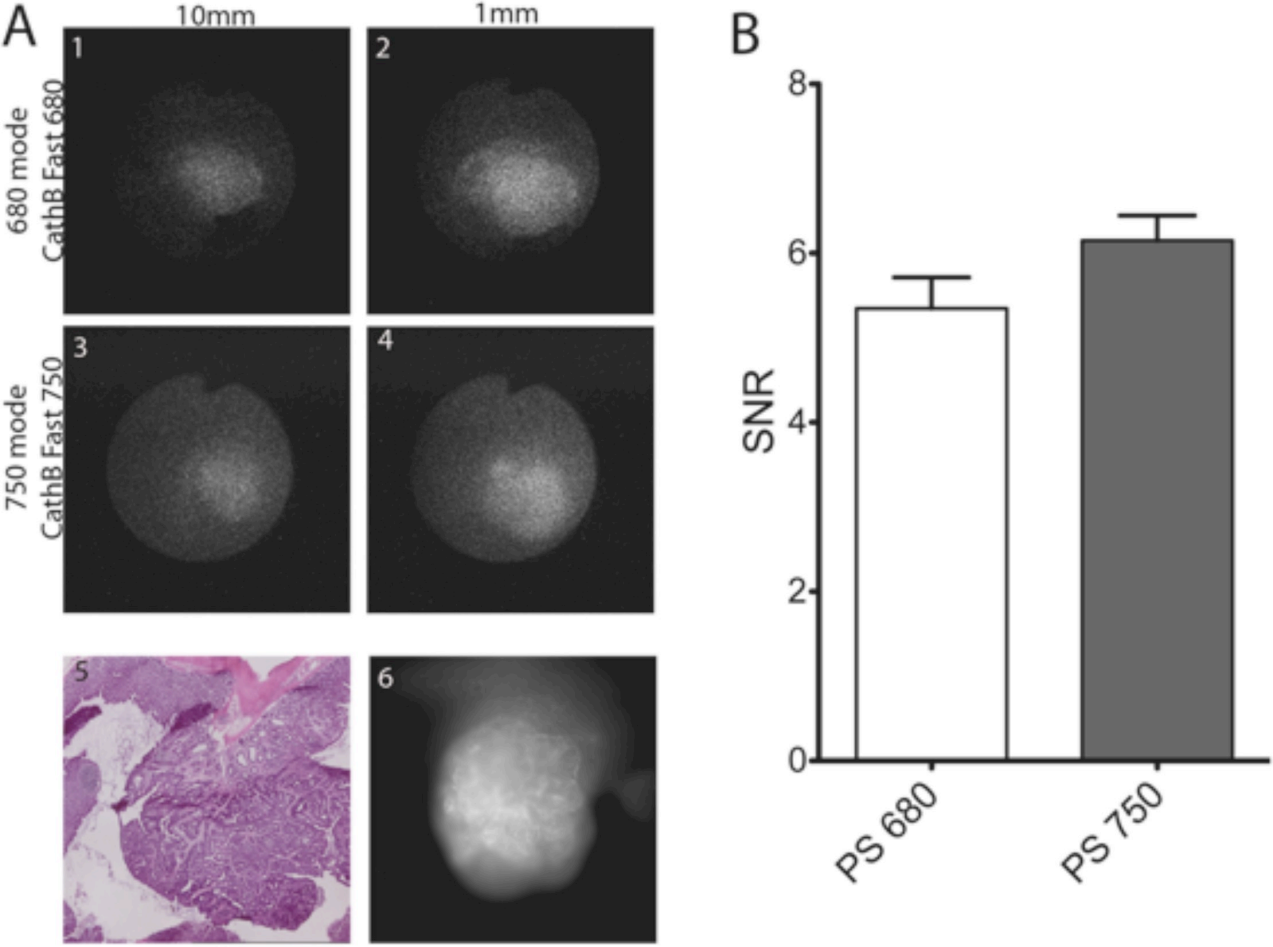
Representative images of colon polyp incubated with both Cath B fast 680 and Cath B fast 750 and imaged with 680 mode (1&2) and 750 mode (3&4) at 10mm (1&3) or 1mm (3&4). The same polyp was imaged for reflectance fluorescence at 680nm (6), and the tissue was stained with H&E (5). The cumulative results of SNR of 6 mice imaged. There is no significant difference of the SNR of the two modes of examination.

In order to verify that polyps can be imaged with equal efficiency in the two modes, we used the same type of probes (SBP) that have the same scaffold, and are activated with the same mechanism, but connected with fluorophores with different optical properties: ProSense 680 (Ex/ Em: 675/694) and ProSense 750 (Ex/ Em: 743/767). The two probes were injected simultaneously (2 nmoles) each mouse each probe in 6 mice and imaged *in vivo* after 18 hours in both 680 and 750 modes and by using the two thirds of the maximum power of both lasers. It should be noted that the areas of high emissions were detected when the tip of the fiberscope was 1 mm of the lesions as well as 10 mm from them **(**compare Fig 4, panel A 1 with 3 and 2 with 4). The high emissions areas correspond to polyp as the H&E image shows (Fig 4, panel A, 5). Similar focal increase in emissions was observed in reflectance fluorescence (Fig 4 panel A, 6). After endoscopy the colon excised and imaged for reflectance fluorescence, and Swiss rolled for necropsy sectioning and H&E staining. The signal to noise ratio (SNR) of the lesions were calculated from the images collected according to materials and methods using the Image J software. (Fig 4). Indeed, the NIRF endoscopic system with NIRF cathepsin activity probes can detect focal fluorescence emissions *in vivo*. H&E staining revealed that it could detect all the polyps in the colon of TS4Cre/cAPC ^+/lox^ mice. Furthermore, all the polyps are visible either in close observation or in distant up to 10 mm.

The signal to noise ratio was at least 5.5-fold in both detection modes. There is no significant variation of the signal to noise ratio (SNR) between the two probes and two modes of detection. **(**SNR of observed polyps was 5.623±0.2299, n = 16, with 680 mode & ProSense 680 while with 750 mode and ProSense 750 was 6.164±0.2343, n = 16; ***P =*** *0*.*1734* unpaired t test with Welch’s correction, non-significant, Fig 4B).

By comparing the images obtained using the 680 or 750 mode, and their intensity (Fig 4 panel A, 1–4, and panel B) it is clear that the system is balanced between the modes of imaging, and useful for controlled comparison of the ability various NIRF probes of cathepsin activity to detect polyps *in vivo*.

### Comparison of substrate based (SBP) and activity based (ABP) NIRF cathepsin activity probes

**Fig 5:**
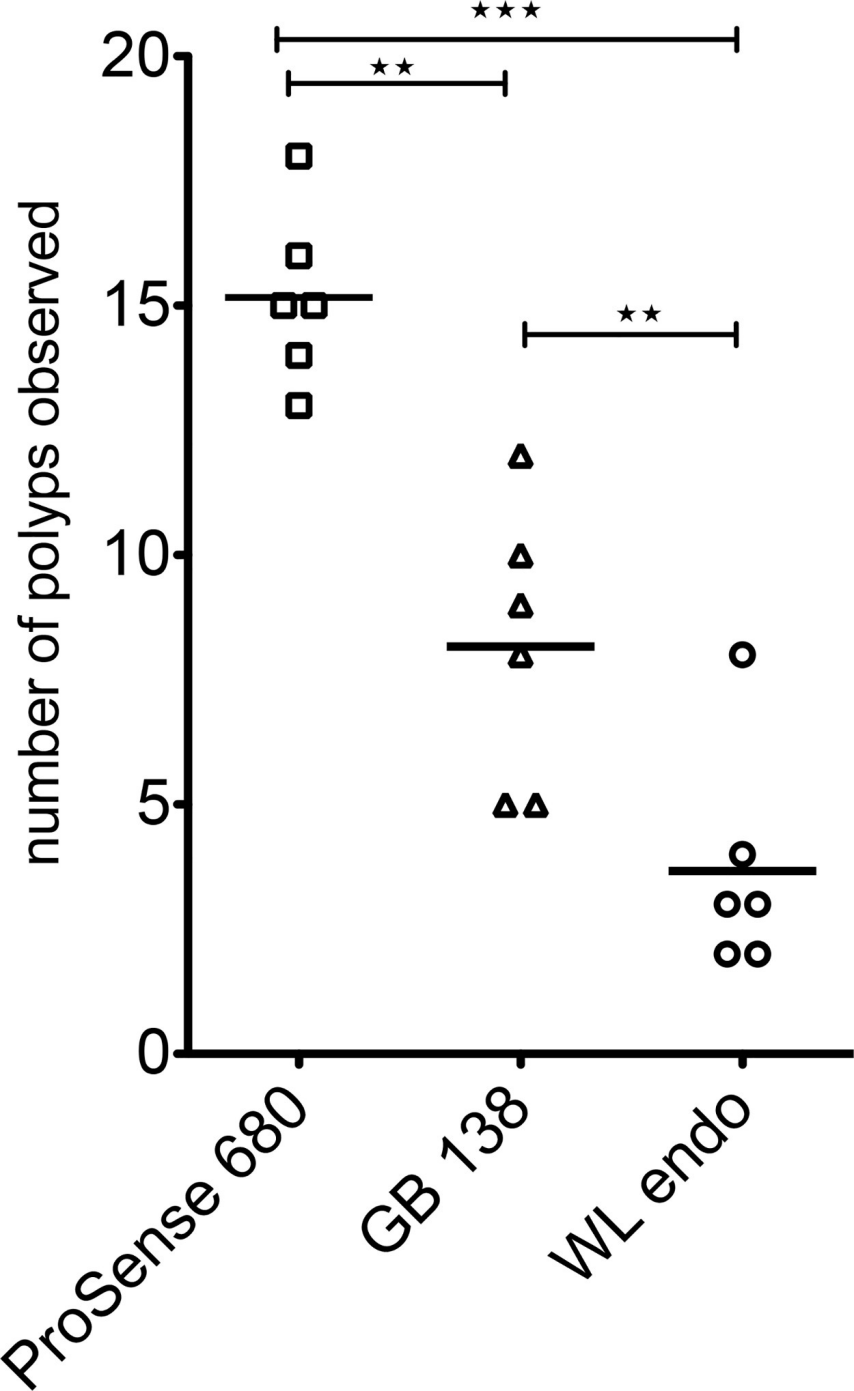
Number of polyps detected in 6 mice injected with both ProSense 680 and GB138 according to materials and methods. The mice were endoscopied with white light (WL endo), 750 mode (GB138) and 680 mode (ProSense 680).

To compare *in vivo* the sensitivity and specificity of NIRF probes developed to detect cathepsin activity, we used ProSense 680 as the standard that it is proven to be efficient to detect most of the lesions in the colon and a NIRF ABP, GB138. GB138 is not quenched, but after 18 hours of staining all the unbound GB138 is removed, and only the molecules linked with the active centers of the active cathepsins remains in the lesions. GB138 is linked with the fluorochrome IRDye 800CW (Ex/ Em: 733 nm/ 792 nm) and can be imaged with the 750 mode [16]. Both probes were injected in 6 TS4Cre/cAPC ^+/lox^ mice. These mice are heterozygous for the floxed APC gene. After the expression of cre gene in colon the floxed APC gene is truncated. Due to loss of heterozygosity dysplastic lesions are developing in these tissues in the form of polyps. The number, and the size of the polyps fluctuates, but all mice develop them at the age of 5 months. We imaged 6 mice in the 680 and 750 mode and using white light endoscopy. The results of this examination are shown in Fig 5. The variation of the numbers observed is statistically significant (WL vs GB138, ***P =* 0.0006,** WL vs ProSense 680 ***P =* 0.00001,** and GB138 vs ProSense 680, ***P =* 0.0002,** unpaired t test two tailed).

### Analysis of the NIRF endoscopy

Observation of the recorded videos revealed that there are small lesions observed with the 680 mode (ProSense 680) which could not be observed using the 750 mode (GB 138, Fig 5A). These lesions were in general small dysplastic lesions that usually appear at the end of the distal colon, close to the rectum.

**Fig 6:**
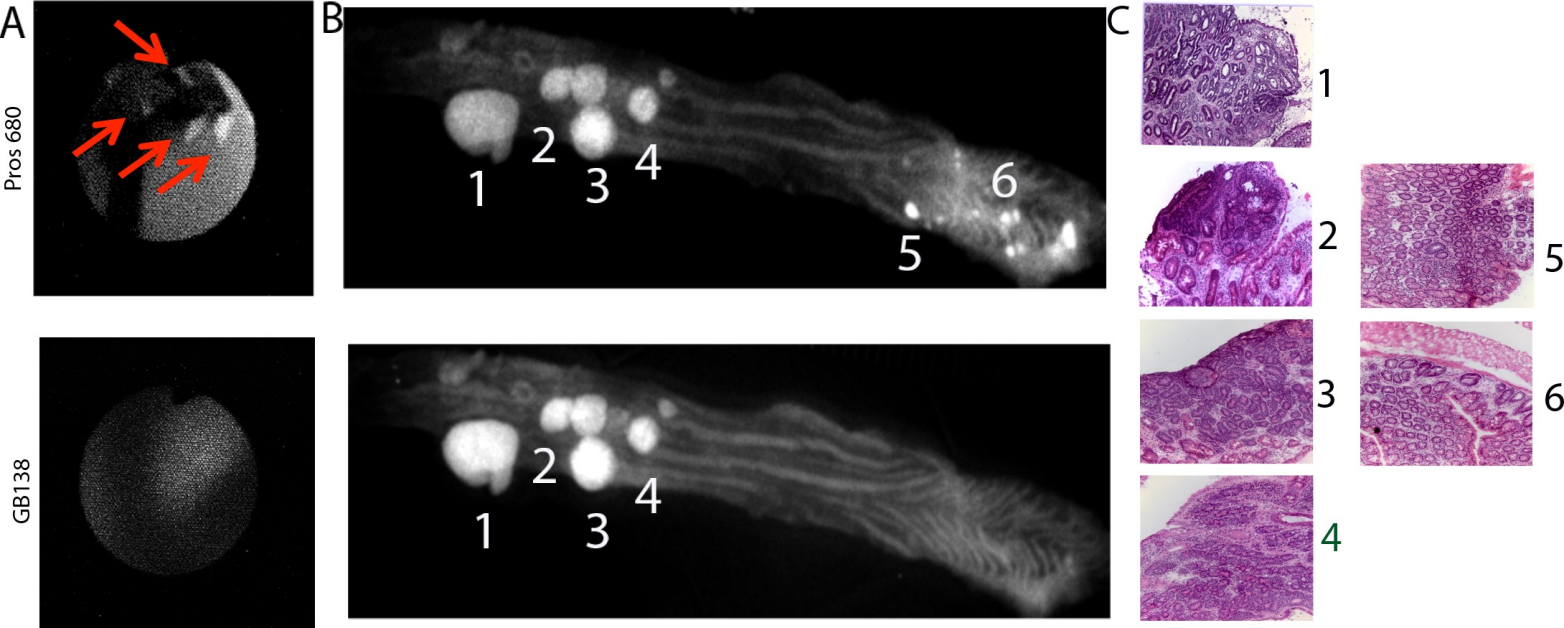
Representative images of mouse colon. A) endoscopic images of the small polyps visible with 680 mode and not with 750 mode. B) flayed open colon imaged with reflectance fluorescence under 660nm and 750 nm light. C) H&E of the areas with high emissions.

To verify these observations, we flayed open the colon of these mice, and imaged them with reflectance fluorescence. Indeed, the observation of the colon under 680 and 750 of the whole colon mounts revealed areas with high emission under the 680 nm which are not visible under the 750 light. These high emissions areas were located at the end of distal colon, close to rectum, and in the same area that were shown under NIRF endoscope. (Fig 6 panel B)

To verify that all the areas with high emissions corresponded to dysplastic lesions, the colons of the six mice were Swiss rolled and the whole roll was sectioned in 5μm sections (~150). Every one out of ten sections stained with H&, and correlated the areas of high emissions with dysplastic lesions. Most of the areas with high emissions were dysplastic, although with the GB138, we could observe some false negatives.

## Discussion

Our system can image autochthonous grown dysplastic lesions in the gastrointestinal tract [11,17], utilizing two modes of detection namely 680 and 750 mode. There is very limited cross talk between the two modes. The cross talk is only possible when the tip of the fiberscope is very close to the lesion and the concentration of the NIRF probes is very high. The two modes are balanced (excitation power is similar).

We have provided an example of a comparison study, showing statistically significant differences between the two probes on their effectiveness to detect polyps.

One of the most important features of this system is that it is possible to utilize various fiberscopes that can access other tissues of the mice, like esophagus and lung in non-invasive mode, as well as, pancreas, prostate and breast tissues laparoscopically. This gives us the possibility to use specific NIRF probes to detect other specific disorders.

We have recently shown that NIRF endoscopy system carries the distinct possibility of becoming a standard procedure to detect early dysplastic lesions in the colon. The system was able to discriminate polyps, and intraepithelial dysplasia in a model of murine colitis. The sensitivity and specificity of the method is exceptional with the ROC curve showing a value of 0.972 (area under the curve) [11]. This system achieved it by using Xe excitation light system and the general cathepsin activity NIRF probe, ProSense 680. Since, we would like to see the usage of this system clinically, we used lasers to increase the penetration of the excitation light into the deep tissues of human colon.

We developed the dual NIRF laser light source for the new NIRF endoscope to address the selection of suitable NIRF cathepsin probes to detect early dysplastic lesions. There is an earlier report which tried to address the problem of comparing the ABPs with SBPs [21]. They used nude mice that were subcutaneously injected with tumor cells. In this report, ABPs look more efficient to detect the tumors. Injected tumors, as compared to the autochthonous tumors, posed problems: A) they grew very quickly without adequate vascular system, B) the center of the tumor is necrotic, and, C) inflammatory myeloid cells were located only in the periphery of the tumors [16]. Whereas, our NIRF endoscopic system is designed to overcome all these problems, as we used mice that grow tumors automatically with tumors that were not necrotic in the center, did not show a marked increase in vascularity, and actually integrated inflammatory myeloid cells into the tumor stroma [10,11,17,23].

The two types of probes we tested have different structure and different end outcome. The SBP (ProSense 680) is a homo polypeptide with many fluorophores on it. Because of the concentration of the fluorophores on each molecule the fluorescence of the probe is quenched. Due to proteolytic activity of cathepsins the polypeptide is hydrolyzed to oligopeptides and their fluorescence becomes unquenched. Furthermore, the oligos can be substrates for cathepsins. This way the emissions of the tissue is catalytically increased. On the other hand, the ABP (GB138) act in a manner as suicide inhibitor. It binds covalently with the active site of active cathepsins in a stoichiometric way, one probe per one active cathepsin. Therefore, the emission of the tissue is lower and specially in small lesions cannot pass the threshold of the electronic noise.

With a kitchen table endoscope, and utilizing one type of SBP and they first proved that the high emissions correspond to dysplastic lesions[23,24]. Several reports have been published for the development of NIRF endoscopic system using a single laser light source [25] using different type of probes. This fact makes them unsuitable to compare NIRF probes in vivo.

Our endoscopic system requires improvement as well. The two examination modes cannot operate simultaneously. The interchange between the two modes is manual and requires switching on a laser, manually changing the excitation and emission filters. Therefore, it is impossible to extrapolate the images obtained by the two-examination modes, a feature that is available in the intravital microscopes.

As far as the probe comparison is concerned, there are several directions we would like to pursue in the future: A) finding the probe that provides the best SNR in detecting colon lesions and allows the possibility to become an investigational new drug (IND) for human studies, B) designing probes that can detect the effectiveness of drugs in treating colon lesions, and C) devising methods to deliver the drug-coated nanoparticles and beads to early dysplastic lesions.

## Supporting information

S1 Fig Specifications of the fiberscopes that could be used with the two-laser excitation light source. (TIF)
