# Correction: Validation of near infrared fluorescence (NIRF) probes *in vivo* with dual laser NIRF endoscope

**DOI:** 10.1371/journal.pone.0210677

**Published:** 2019-01-07

**Authors:** Manisha Shrivastav, Elias Gounaris, Mohammad W. Khan, Jeffrey Ko, Stacy H. Ryu, Matthew Bogyo, Andrew Larson, Terrence A. Barrett, David J. Bentrem

The eighth author’s name is spelled incorrectly. The correct name is: Terrence A. Barrett.
