# Supplementary figures and images for: Validation of near infrared fluorescence (NIRF) probes in vivo with dual laser NIRF endoscope
Source: PLoS One. 2018 Nov 2;13(11):e0206568. doi: 10.1371/journal.pone.0206568 (PMC6214553; doi:10.1371/journal.pone.0206568)

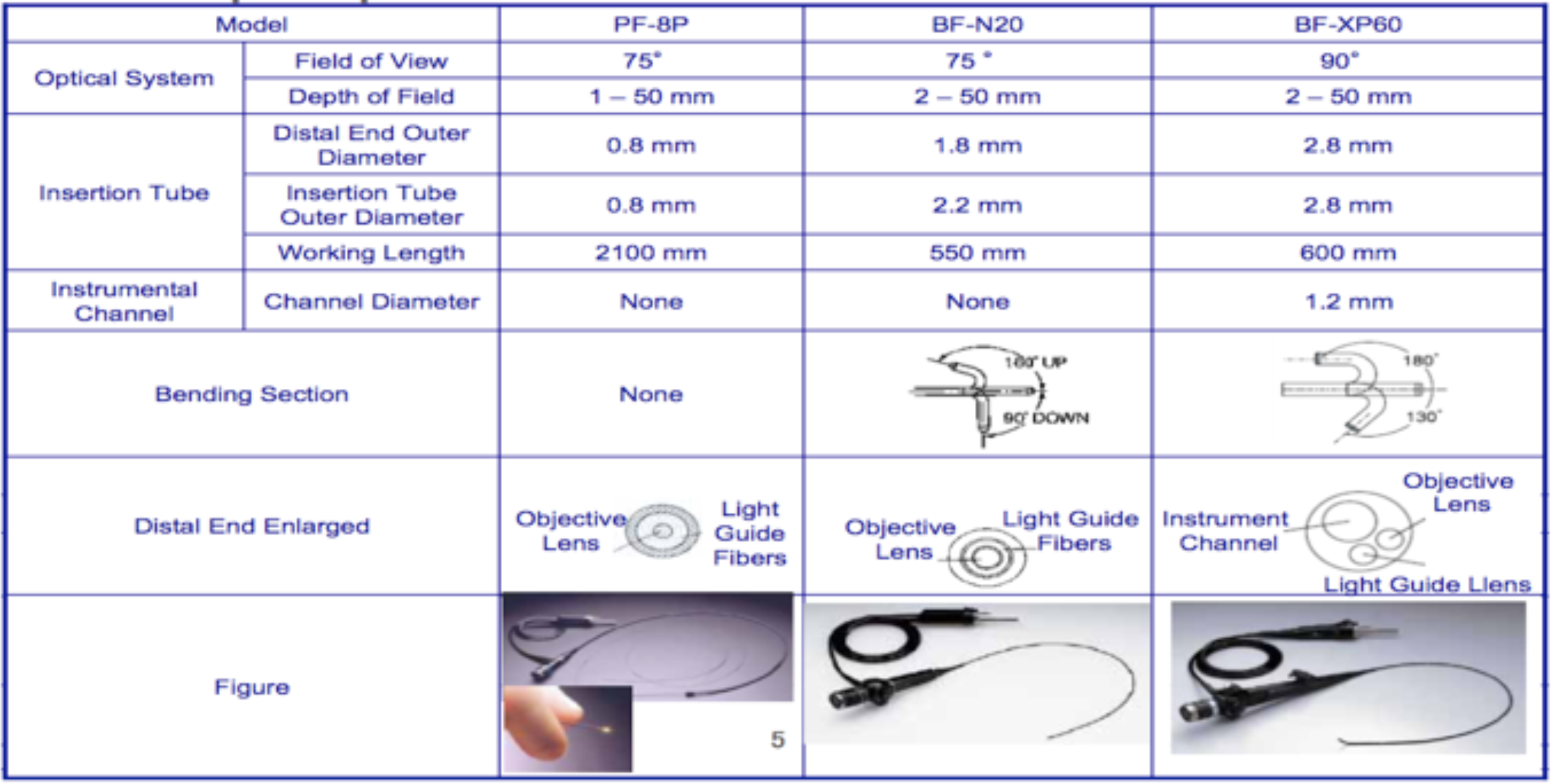

Supplement: S1 Fig — (TIF) [file pone.0206568.s001.tif]
